# Correlates of healthy life expectancy in low- and lower-middle-income countries

**DOI:** 10.1186/s12889-018-5377-x

**Published:** 2018-04-11

**Authors:** Md Shariful Islam, Md Nazrul Islam Mondal, Md Ismail Tareque, Md Aminur Rahman, Md Nazrul Hoque, Md Munsur Ahmed, Hafiz T. A. Khan

**Affiliations:** 1 Department of Population Science and Human Resource Development, University of Rajshahi, Rajshahi, 6205 Bangladesh; 2 Centre for Ageing Research and Education, Duke-NUS Medical School, Singapore, 169857 Singapore; 3 Hobby Center for Public Policy, University of Houston, Houston, TX USA; 4 Social Marketing Company, Dhaka, 1213 Bangladesh; 5 The Graduate School, University of West London, St Mary’s Road, Ealing, London, W5 5RF UK

**Keywords:** Healthy life expectancy, Low- and lower-middle-income countries, Quality of life, Correlates of healthy life expectancy

## Abstract

**Background:**

Healthy life expectancy (HALE) at birth is an important indicator of health status and quality of life of a country’s population. However, little is known about the determinants of HALE as yet globally or even country-specific level. Thus, we examined the factors that are associated with HALE at birth in low- and lower-middle-income countries.

**Methods:**

In accordance with the World Bank (WB) classification seventy-nine low- and lower-middle-income countries were selected for the study. Data on HALE, demographic, socioeconomic, social structural, health, and environmental factors from several reliable sources, such as the World Health Organization, the United Nations Development Program, Population Reference Bureau, WB, Heritage Foundation, Transparency International, Freedom House, and International Center for Prison Studies were obtained as selected countries. Descriptive statistics, correlation analysis, and regression analysis were performed to reach the research objectives.

**Results:**

The lowest and highest HALE were observed in Sierra Leone (44.40 years) and in Sri Lanka (67.00 years), respectively. The mean years of schooling, total fertility rate (TFR), physician density, gross national income per capita, health expenditure, economic freedom, carbon dioxide emission rate, freedom of the press, corruption perceptions index, prison population rate, and achieving a level of health-related millennium development goals (MDGs) were revealed as the correlates of HALE. Among all the correlates, the mean years of schooling, TFR, freedom of the press, and achieving a level of health-related MDGs were found to be the most influential factors.

**Conclusion:**

To increase the HALE in low- and lower-middle-income countries, we suggest that TFR is to be reduced as well as to increase the mean years of schooling, freedom of the press, and the achievement of a level of health-related MDGs.

## Background

Healthy life expectancy (HALE) at birth is the summary measure of a population’s health, developed by the World Health Organization (WHO), which attempts to capture a complete estimate of health than life expectancy (LE). HALE is an estimate of how many years that a person might live in a healthy state. It is an indicator of health conditions of the country’s population, including the impacts of mortality and morbidity. It is related to the declined rate of mortality and morbidity and an improvement of health measures since the 1960’s [1]. In 1964, Sanders published important results focusing on measuring community health levels [2]. In 1969, an improved measure of the health, health-adjusted life expectancy or HALE, was published by the United States (US) Department of Health, Education, and Welfare for the first time [1]. But, the methods for calculating HALE were publicized by Sullivan in 1971 [2].

Different demographic, socioeconomic, social structural, health-related and environmental factors, as well as the regional variation, found to be the influential factors of HALE [3]. The lowest HALE has been reported in African region (52.30 years) while it is around 68.00 years in America and Europe and 60.50 years in South Asia [3]. Overall, the global HALE is estimated to be 63.10 years in 2015 [3]. Beside these geographical variations, variation is also observed by the economic status of the different countries. Lower HALE is observed in the low-income countries (53.00 years) which are around 17.00 years higher in the high-income countries (70.00 years). This difference is around 4 years between low- and lower-middle-income countries [3]. There are many factors that often created to increase HALE. Increasing mean years of schooling [4], health expenditure, physician density, entrance to improved drinking-water sources and hygienic sanitation system, economic freedom [5], freedom of the press, corruption perceptions index (CPI), achieving a level of health-related millennium development goals (MDGs), and gross national income (GNI) per capita; and decreasing the human immunodeficiency virus (HIV) prevalence rate [6], total fertility rate (TFR), carbon dioxide (CO_**2**_) emission rate, and prison population rate significantly contribute to increasing the average HALE at birth. But these factors may vary from one geographic region to the others which need to be examined. Robine and Ritchie reviewed and evaluated the usefulness of HALE as a global indicator of changes in a population’s health [7]. Saito and others provided a brief overview of health expectancy and the issues to be considered in operationalizing and interpreting health expectancy. They introduced the concept of health expectancy, discussed the measures used to compute health expectancy, and methods of calculation [8]. Mathers and others displayed the global patterns of HALE in the year 2002 [9]. Several studies are conducted on HALE, viz., Summary measures of population health: methods for calculating HALE [1]; HALE [2]; HALE: comparison of OECD countries in 2001 [10]; Health-Adjusted Life Expectancy [11]; A comparison of self-rated health, health status, and health promotion behaviors between low- and non-low-income elderly women [12]; HALE and the correlates of self-rated health in Bangladesh in 1996 and 2002 [13]; HALE and the correlates of self-rated health in an ageing population in Rajshahi district of Bangladesh [14]. However, no study has been conducted to identify the determinants of HALE with a specific focus on the economic status or income groups. Therefore, the present study is conducted to identify the influential factors that are associated with HALE in low- and lower-middle-income countries by using the secondary data. The findings may help the policy-makers and researchers to determine the influential factors of HALE in low- and lower-middle-income countries and thereby take appropriate measures as to how to raise HALE in these countries.

## Methods

### Data

Most of the variables that had the significant effects on HALE in the previous studies were selected for this study. Data on low- and lower-middle-income countries were obtained from the specialized agencies of the United Nations (UN) systems. The UN agencies rely on an extensive peer review process, which is conducted through leading regional and national statistics offices and international organizations, thus ensuring the highest level of data consistency and accuracy. Seventy-nine countries were classified as low- and lower-middle-income countries by the World Bank (WB) (see Appendix A). Several indicators of HALE including HLAE for these 79 countries were obtained from several sources, e.g., WHO [3, 15], United Nations Development Program (UNDP) [16], Population Reference Bureau (PRB) [17], WB [18], Transparency International (TI) [19], Heritage Foundation (HF) [20], Freedom House (FH) [21], and International Center for Prison Studies [22].

### Dependent variable

HALE at birth is considered as the dependent variable, which is the measurement of how many years that a person might live in a healthy state.

### Independent variables

Different demographic, socioeconomic, social structural, health-related, and environmental factors were considered as the independent variables. Demographic variable includes the TFR. Socioeconomic variables include mean years of schooling, GNI per capita, and health expenditure. Social structural variables encompass the freedom of the press, CPI, prison population rate, and economic freedom. Worldwide known HIV prevalence rate, achieving a level of health-related MDGs, and physician density are considered as the health-related factors. Environmental factors include improved drinking-water sources using rate, improved sanitation using rate, and CO_**2**_ emission rate per capita (tonnes) (for details see Appendix B). A level of health-related MDGs was calculated using the following ten variables or targets named: percent reduction in under-five mortality rate, 1990–2013 (*T* = 67); Measles immunization coverage among 1 year old (%), 2013 (*T* = 90); percent reduction in maternal mortality ratio, 1990–2013 (*T* = 75); births attended by skilled health personnel (%) (T = 90); antenatal care coverage (%): at least one visit (*T* = 100); unmet need for family planning (%) (*T* = 0); percent reduction in HIV incidence, 2001–2013 (T= > 0); percent reduction in mortality rate of tuberculosis (among HIV-negative people), 1990–2013 (T= > 50); percent reduction in proportion of population without access to improved drinking-water sources, 1990–2012 (*T* = 50); percent reduction in proportion of population without access to improved sanitation, 1990–2012 (T = 50). The extent of progress for a country has been classified into three categories named: (i) met or on track, (ii) substantial progress, and (iii) noo or limited progress [see [15] for details]. Each of the above targets is labeled as ‘1’ when it satisfies the ‘met or on track’ category of the achievement progress of the MDGs; otherwise, it is labeled as ‘0’. Then the row total was performed to get an achieving level of health-related MDGs for a country.

### Statistical analyses

Descriptive statistics were used to describe the situations of all low- and lower-middle-income countries. After this, the Pearson’s correlation analysis was performed to see the relationships among the selected variables. To examine the effects of the independent variables on the dependent variable, several multiple linear regression models were fitted. Next multicollinearity problem was checked in the regression analyses by examining the tolerance values. The tolerance values less than 0.40 indicate a strong multicollinearity [23], and there was a strong multicollinearity among physician density, TFR, GNI per capita, and health expenditure. Therefore, all the collinear variables were dropped one by one except TFR because it is very close to 0.40. And finally, a regression analysis was performed to identify the most influential factors that are associated with HALE. Here noted that the logarithm values of the two variables were used which were GNI per capita and physician density. The entire analysis of the study was done with the statistical software Stata /MP Version 13 (Stata Corporation LP, College Station, Texas; USA). The HALE of the study countries is presented with the help of the geographical software ArcGIS 9.3.

## Results

**Fig. 1:**
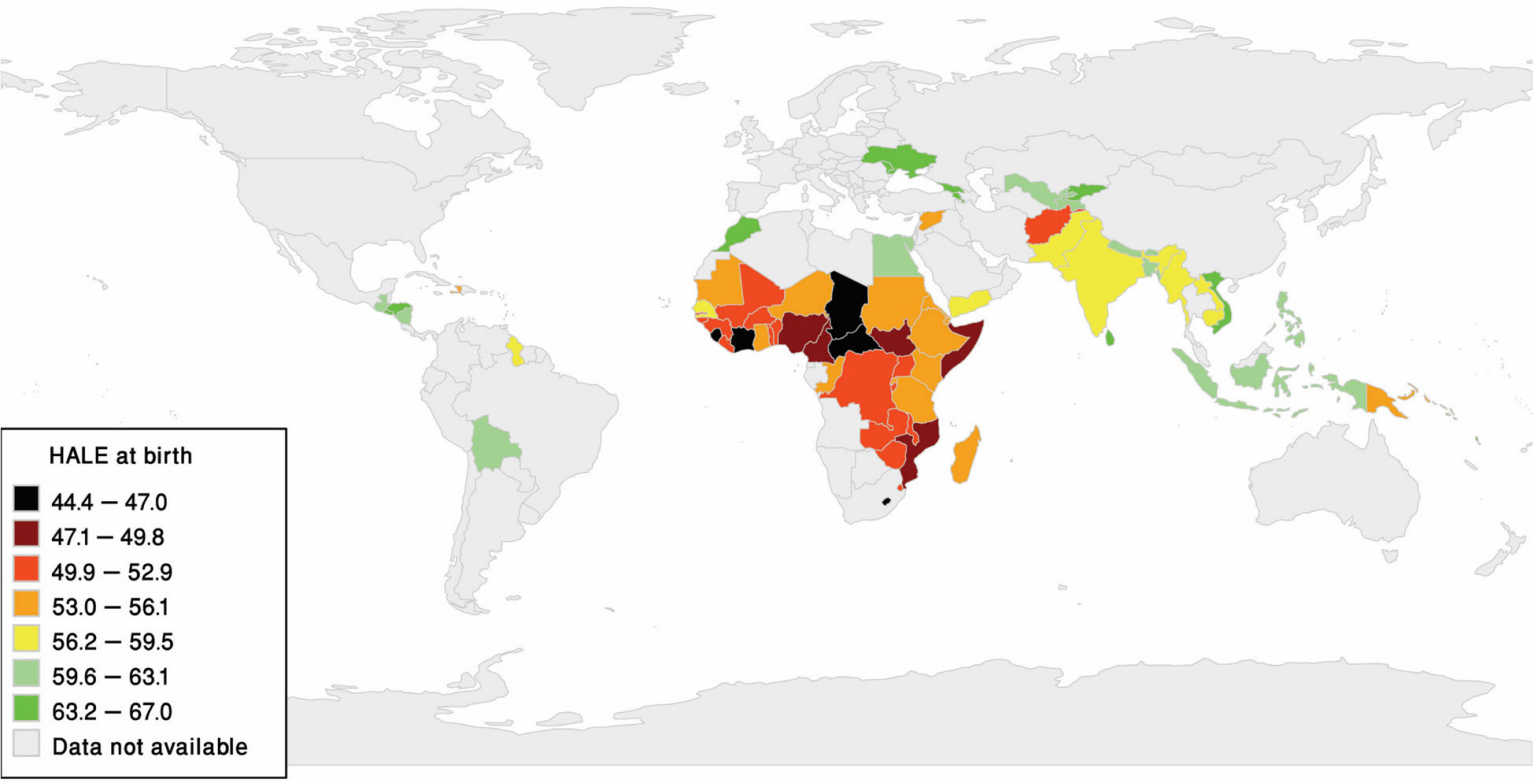
Healthy life expectancy at birth for the study countries

**Fig. 2:**
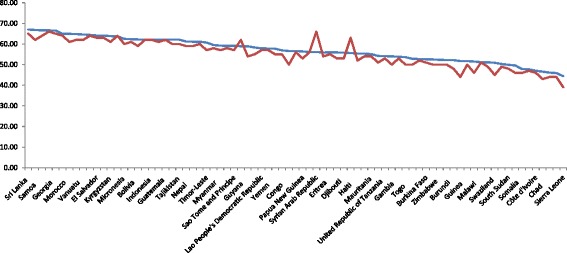
Trend of healthy life expectancy at birth in two data points for the study countries

Table 1 presents the current situation of HALE (see Fig. 1) and associated factors in 79 low- and lower-middle-income countries. Fig. 2 shows the HALEs of the countries for two-time points (2013 and 2015 years). The HALEs of these countries are arranged in descending order. The HALEs of these countries are seen increased in 2015 compared to the year 2013. The HALE, mean years of schooling, health expenditure, physician density, improved sanitation using rate, freedom of the press, economic freedom, and achieving a level of health-related MDGs are observed very low among the African countries. On the other hand, the highest HALE, mean years of schooling, health expenditure, physician density, improved drinking-water sources using rate, improved sanitation using rate, freedom of the press, CPI, economic freedom, and achieving a level of health-related MDGs are seen in the Asian countries. The GNI per capita, prison population rate, and CO_**2**_ emission rate per capita (tonnes) are found very low among the African countries. On the other hand, the highest GNI per capita, and prison population rate are seen in the American countries. And the CO_**2**_ emission is found the highest among the European countries. The lowest HALE is seen in Sierra Leone (44.40 years) and the highest HALE is seen in Sri Lanka (67.00 years). The lowest mean years of schooling are found in Burkina Faso (only1.10 years) and the highest mean years of schooling are found in Kyrgyzstan (13 years). The TFR is seen very low in the European countries. In the Republic of Moldova, it is only 1.30 births per woman. In the Asian countries, the HIV prevalence rate is seen very low than other countries except for Egypt and Morocco. The lowest value of HIV prevalence rate is 0.10, i.e., there is only one HIV-infected person per thousand people in Egypt, Morocco, Bangladesh, Bhutan, Pakistan, Philippines, Sri Lanka, and Yemen. Again, the highest TFR (7.60 in Nigeria, 6.20 in Mali) and HIV (27.70% in Swaziland, 23.40% in Lesotho, 16.70% in Zimbabwe) prevalence rates are seen among the African countries. On the other hand, in Uzbekistan, the freedom of the press is very low (5.00) and in Micronesia, the freedom of the press is the highest (79.00) among the other low- and lower-middle-income countries. In the case of achieving a level of health-related MDGs, Comoros and South Sudan (0.00) hold the lowest position and Viet Nam (8.00) hold the highest position. Among the low- and lower-middle-income countries, lowest and highest urban populations were found in Burundi (12.36%) and Djibouti (77.43%), respectively.

**Table 1:** Descriptive statistics of the selected variables for the low- and lower- middle income countries

Variables	*N*	Mean	Median	Standard deviation	MinimumValue	Country	MaximumValue	Country
Healthy life expectancy	79	57.05	56.60	5.81	44.40	Sierra Leone	67.00	Sri Lanka
Mean years of schooling^a^	79	5.60	5.10	2.67	1.10	Burkina Faso	12.50	Kyrgyzstan
GNI per capita^a^	79	1795.08	1400.00	1307.05	127.90	Somalia	6930.00	Guyana
Health expenditure^a^	79	101.87	76.00	82.69	0.00	Somalia	415.00	Micronesia
Total fertility rate^a^	79	3.90	4.00	1.38	1.30	Republic of Moldova	7.60	Niger
Physician density^b^	73	5.65	1.80	9.05	0.10	Liberia	42.70	Georgia
CO_2_ emission rate^c^	79	0.77	0.40	0.98	0.00	Burundi and Chad	6.30	Ukraine
HIV prevalence rate^a^	72	2.59	0.75	4.95	0.10	Afghanistan, Egypt, Morocco, Bangladesh, Bhutan, Pakistan, Philippines, Sri Lanka, Syrian Arab Republic, and Yemen	27.70	Swaziland
Improved drinking-water sources using rate	79	77.97	79.00	15.98	32.00	Somalia	100.00	Armenia, Bhutan and Georgia
Improved sanitation using rate	79	47.14	42.00	27.16	7.00	South Sudan	100.00	Uzbekistan
Freedom of the press	79	41.73	42.00	18.13	5.00	Uzbekistan	79.00	Micronesia
Corruption perceptions index	79	30.49	30.00	10.21	8.00	Somalia	65.00	Bhutan
Prison population rate	75	110.72	84.00	89.12	16.00	Central African Republic	517.00	El Salvador
Economic freedom	74	54.70	54.80	6.40	37.60	Zimbabwe	73.00	Georgia
Achieving a level of health-related MDGs	79	3.20	3.00	1.71	0.00	Comoros and South Sudan	8.00	Viet Nam
Urban population (% of total)	78	39.98	39.30	15.79	12.36	Burundi	77.43	Djibouti

Notes: ‘*N* Number of countries’, ‘*GNI* Gross National Income’, ‘*CO*_2_ Carbon dioxide’, ‘*HIV* Human Immunodeficiency Virus’, ‘*MDGs* Millennium Development Goals’, ‘^ a^, 2014’; ‘^b^, 2007–2013’; ‘ ^c^2011’

Table 2 shows the significant positive relationships of HALE with mean years of schooling (*r* = 0.58, *p* < 0.01), GNI per capita (*r* = 0.65, *p* < 0.01), health expenditure (*r* = 0.58, *p* < 0.01), physician density (*r* = 0.70, *p* < 0.01), CO_**2**_ emission (*r* = 0.50, *p* < 0.01), improved drinking-water sources using rate (*r* = 0.62, *p* < 0.01), sanitation using rate (*r* = 0.75, *p* < 0.01), CPI (*r* = 0.31, *p* < 0.01), prison population rate (*r* = 0.44, *p* < 0.01), and economic freedom (*r* = 0.32, *p* < 0.01), and achieving a level of health-related MDGs (*r* = 0.50, *p* < 0.01). On the other hand, significant negative relations are found of TFR (*r* = − 0.75, *p* < 0.01) and HIV prevalence rate (*r* = − 0.43, *p* < 0.01) with HALE in the low- and lower-middle-income countries of the world.

**Table 2:** Pearson’s correlation coefficients between the selected variables

	*Y*	*X*_*1*_	*X*_*2*_	*X*_*3*_	*X*_*4*_	*X*_*5*_	*X*_*6*_	*X*_*7*_	*X*_*8*_	*X*_*9*_	*X*_*10*_	*X*_*11*_	*X*_*12*_	*X*_*13*_	*X*_*14*_	*X*_*15*_
*Y*	1															
*X*_*1*_	0.58**	1														
*X*_*2*_	0.65**	0.55**	1													
*X*_*3*_	0.58**	0.58**	0.75**	1												
*X*_*4*_	−0.75**	−0.57**	−0.65**	−0.54**	1											
*X*_*5*_	0.70**	0.71**	0.63**	0.50**	−0.73**	1										
*X*_*6*_	0.50**	0.59**	0.55**	0.49**	− 0.59**	0.64**	1									
*X*_*7*_	−0.43**	0.02	− 0.06	0.04	0.15	−0.28*	− 0.11	1								
*X*_*8*_	0.62**	0.44**	0.51**	0.51**	−0.62**	0.56**	0.48**	−0.11	1							
*X*_*9*_	0.76**	0.65**	0.60**	0.53**	−0.72**	0.82**	0.65**	−0.21	0.65**	1						
*X*_*10*_	0.16	0.08	0.28*	0.33**	−0.02	−0.12	− 0.02	− 0.02	0.22*	− 0.16	1					
*X*_*11*_	0.31**	0.14	0.35**	0.35**	−0.26*	0.05	0.11	0.13	0.51**	0.22*	0.42**	1				
*X*_*12*_	0.44**	0.38**	0.42**	0.56**	−0.49**	0.39**	0.28*	0.10	0.3**	0.52**	0.03	0.44**	1			
*X*_*13*_	0.32**	0.18	0.25*	0.29*	−0.22	0.27*	0.03	− 0.13	0.37**	0.31**	0.24*	0.60**	0.46**	1		
*X*_*14*_	0.50**	0.27*	0.22	0.28*	−0.50**	0.31**	0.22	−0.11	0.52**	0.43**	−0.02	0.37**	0.45**	0.32**	1	
*X*_*15*_	0.18	0.13	0.29*	0.29*	−0.26*	0.31**	0.32**	−0.25*	0.29*	0.21	0.04	0.16	0.15	0.14	0.09	1

Notes: ‘*, *p* < 0.05, and **, *p* < 0.01’; ‘*Y*, Healthy life expectancy’; ‘*X*_*1*_, Mean years of schooling’; ‘*X*_*2*_, Gross national income per capita’; ‘*X*_*3*_, Health expenditure’; ‘*X*_*4*_, Total fertility rate’; ‘*X*_*5*_, Physician density’; ‘*X*_*6*_, Carbon dioxide emission rate’; ‘*X*_*7*_, Human immunodeficiency virus prevalence rate’; ‘*X*_*8*_, Improved drinking water sources using rate’; ‘*X*_*9*_, Improved sanitation using rate’; ‘*X*_*10*_, Freedom of the press’; ‘*X*_*11*_, Corruption perceptions index’; ‘*X*_*12*_, Prison population rate’; ‘*X*_*13*_, Economic freedom’; ‘*X*_*14*_, Achieving a level of health-related Millennium Development Goals’; ‘*X*_*15*_, Urban population (% of total)’

**Fig. 3:**
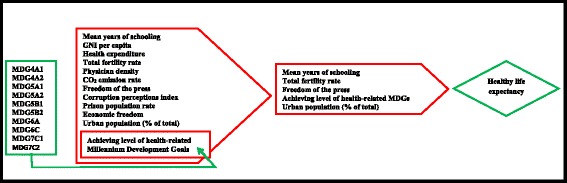
Correlates of healthy life expectancy at birth

Table 3 represents the results of multiple linear regression models. The regression analyses identified the mean years of schooling, TFR, HIV prevalence rate, CO_**2**_ emission rate, freedom of the press, CPI, and prison population rate, economic freedom, and achieving a level of health-related MDGs are as the correlates of HALE. The multiple regression model (Model 1) (${R}_a^2=0.61$) gives the most influential factors that are associated with HALE which are mean years of schooling, TFR, freedom of the press, and achieving a level of health-related MDGs (see Fig. 3). Among all these correlates the mean years of schooling, freedom of the press, and achieving a level of health-related MDGs have shown positive effects and only the TFR has shown negative effects on HALE. The other variables which were included in the analysis have shown the general relations with outcome variable, HALE. So, we can say that among all the independent variables the higher mean years of schooling, freedom of the press, and achieving a level of health-related MDGs; and the lower TFR are the most influential factors which increase the nation’s average HALE.

**Table 3:** Multiple linear regression models explaining the healthy life expectancy

Explanatory Variables	Model 1		Model 2		Model 3		Model 4		Model 5		Model 6	
	Adjusted β	SE of β (95% CI)	Unadjusted β	SE of β (95% CI)	Unadjusted β	SE of β (95% CI)	Unadjusted β	SE of β (95% CI)	Unadjusted β	SE of β (95% CI)	Unadjusted β	SE of β (95% CI)
Mean years of schooling	0.46	0.19* (0.09, 0.85)	1.25	0.20** (0.85, 1.66)								
Total Fertility Rate	−2.26	0.42** (−3.09, −1.43)			−3.14	0.32** (−3.77, −2.50)						
Freedom of the Press	0.05	0.02* (−0.002, 0.09)					0.05	0.04 (−0.02, 0.12)				
Achieving a level of health-related MDGs	0.60	0.28* (0.04, 1.17)							1.69	0.34** (1.03, 2.36)		
Urban population (% of total)	−0.004	0.03 (−0.06, 0.05)									0.07	0.04 (−0.02, 0.15)
${R}_a^2$	0.61		0.32		0.55		0.01		0.24		0.02	

Notes: ‘*β* Regression coefficient’, ‘*SE* Standard error’, ‘*CI* Confidence interval’, ‘** Significant at *p* < 0.01’; ‘* Significant at *p* < .05’; ‘*MDGs* Millennium Development Goals’

## Discussion

In this first representative study of low- and lower-middle-income countries, an attempt is made to identify the correlates that are associated with HALE. We found the mean years of schooling, TFR, freedom of the press, and achieving a level of health-related MDGs as the significant correlates of HALE. The effects of other independent variables on HALE are statistically insignificant.

The level of education of the respondents is an important factor of HALE. The findings of this study are consistent with the results of other studies conducted in developed and developing countries [4, 24]. This may be due to the fact that the higher education levels are typically related with well-timed receiving healthcare, and also more awareness about the health. The higher rate of receiving prenatal care, optimize the use of maternal healthcare services also found to be the higher among the educated individuals which are found as the influential factors of developing HALE. We know that the educated individuals earn higher real wages. The higher real wages indicate the high average household income and enabling people to enlarge the quality and quantity of the purchased healthcare services. Additionally, educated people have knowledge about proper nutrition, hygiene, and healthcare services, as well as common illness-preventative measures [25] and thus they seem to enhance their HALE. So, we see that HALE increases due to increase in mean years of schooling. A study conducted in the United Nations of America (USA) also supported that the increasing mean years of schooling is an important factor of widening the HALE [4]. Valkonen et al. also supported by saying that the LE, as well as disability-free life expectancy (HALE), showed systematic relationships with the level of education: the higher the level of education, the higher LE and disability-free life expectancy [26]. This finding is also supported by Deka and Nath [27], and Shin et al. [12]. Thus, it is theorized that as mean years of schooling increases, average HALE will increase.

The coefficients for TFR and HIV prevalence rate are statistically significant and shown negative effects on HALE in all the regression models. The TFR of Nigeria and Mali are 7.60 and 6.20 respectively whereas it is on an average 1.60 in the high-income countries [18]. The increase in an average number of births to a woman means the decrease in HALE. Mondal and Shitan suggested that increases in TFR are likely to decrease average LE in a country [28]. As the TFR decreases the average LE so the HALE is also decreasing due to the TFR. Hence it is needed to reduce the TFR to maximize the average HALE of the population. HIV has become a major public health problem in many countries and one of the world’s most serious health and development challenges. Eventually, the HIV attacks the immune system of the infected individual which is a non-curable virus. Without treatment, the net median survival time with HIV is 9–11 years [29], meaning that individuals who have tested positive for HIV face a drastically reduced lifespan. A greater percentage of infected adults could also mean higher HIV transmission rates to children. This virus reduces a country’s average HALE. The HIV prevalence rate was identified as a responsible factor of lowering HALE by the authors in the previous studies, like [6]. HIV prevalence rate is likely to decrease average LE due to the increase in HIV prevalence [28]. As the HIV prevalence rate decreases the average LE, so we can say that the HIV prevalence rate also decreases the HALE at birth. Thus, it is seen that as the percentage of HIV-infected adult increases, average HALE will decrease. So, it is needed to control the HIV prevalence rate to maximize the average HALE of the population. Greater health expenditure, physician density, improved drinking-water sources using rate, freedom of the press, and CPI mean the greater HALE. Thus, it is hypothesized that if the values of these variables increase, the average HALE will be increased. On the other hand, if both the CO_**2**_ emission rate and prison population rate decrease, the average HALE at birth will increase. The above findings have significant effects on increasing HALE. HALE increases throughout the countries at a more rapid rate than LE, advising to reduce the disability for reducing mortality [6]. Wolfson, and Tareque et al. also supported by saying that HALE increases more rapidly than LE [11, 13].

Another measure of a country’s standard of living is freedom of the press, and obviously, it is related to HALE. The freedom of communication and expression through various mediums like electronic media and published materials is known as the freedom of the press or freedom of the media. In the twentieth century, Liebling, an American journalist, was excellently abridged the idea of “freedom of the press”, who wrote, “Freedom of the press is guaranteed only to those who own one” [30]. In which place, it exists mostly implies the absence of interference from the government and other powerful organizations; it is maintained through the constitution or other legal protections. Freedom House, the US-based non-governmental organization, is published a yearly report on freedom of the press by measuring the level of freedom and editorial independence enjoyed by the press in every nation and significant disputed territories around the world. Levels of freedom are scored on a scale from 0 (most free) to 100 (least free) [21]. But we converted this scale to 100 (most free) to 0 (least free). Depending on the basics, the nations are then classified as “Free”, “Partly Free”, or “Not Free”. The impact of freedom of the press on HALE has never been measured. It is difficult, though not impossible, to calculate such impact. However, freedom of the press always has a positive influence on HALE. To achieve the high HALE, and MDGs a freer press has a great contribution [31]. In 2015, the countries where the press was the freest were Micronesia, Vanuatu, Solomon Islands, Cabo Verde, and Sao Tome and Principe [21], and in these countries, the HALE is high than others [3]. The country with the least degree of press freedom was Uzbekistan, Eritrea, Syrian Arab Republic, Gambia, and Sudan [21], and in these countries, the HALE is low than others [3]. By promoting the level of freedom of the press, Government and international organizations provide a powerful development tool. A free press establishes an instrument of development, as such in the same way as education or investment, which promotes the HALE of the country’s population [30]. Thus, it is concluded that as the level of freedom of the press increases, average HALE will increase.

One of the most important factors of a country’s standard of living is achieving a level of health-related MDGs which is highly significant with HALE and has the great influences on HALE [15]. The MDGs are eight international development goals that all 194 UN member states and at least 23 international organizations have agreed to achieve by the year 2015 [15]. In 2015, the MDGs have come to the end of their term. Progress towards the MDGs, on the whole, has been remarkable. Country progress towards the achievement of the health-related MDGs and targets has also been considerable. During the MDG era, many of the health-related MDGs were achieved, with the corresponding targets. The results of this study demanded that the HALE is low in those African countries where the achieving a level of health-related MDGs is observed very low, and the HALE is high in those Asian countries where the achieving a level of health-related MDGs is observed very high [15]. In a study, Lomazzi and others also supported that the achieving a level of health-related MDGs has a positive impact on HALE [32]. Thus, it is assumed that as the achieving a level of health-related MDGs increases, average HALE will increase.

A limitation of this work is that we only studied the data for the most common affecting factors, i.e., those factors which are found to be significantly related to HALE. We did not consider the mortality-related factors which will be our next study. Also, the study is limited to the low- and lower-middle-income countries. Again, the sources and quality of data are different in different countries. Some low- countries have complete civil registration and vital statistics and regular censuses of the entire population as the data sources. On the other hand, many lower-middle-income countries have an incomplete birth and death registration systems as well as the lack of continuous realistic data on mortality and HALE. But, all data which are analyzed in this study collected from the very reliable sources.

## Conclusions

An investigation is made to find out the main powerful factors affecting HALE from the demographic variables, socioeconomic status, social structural indicators, health-related factors and environmental issues which have the significant effects on HALE in the low- and lower-middle-income countries. The study signifies that among all the associated factors, mean years of schooling, TFR, freedom of the press, and achieving a level of health-related MDGs are the principal factors which have the most important effects on a nation’s average HALE. Our results have some policy implications for these countries, especially those in Africa. Urgent action is necessary to enhance HALE. The national and international efforts should be designed at increasing average HALE to raise the awareness about the mean years of schooling, TFR, freedom of the press, and achieving a level of health-related MDGs among the country’s population. By setting up a required number of schools the whole nation has to invest all its energies to ensure quality primary education for all for the extension of universal primary education to enhance the education level of a country. The viable options to lower the TFR are to develop education system, the family planning programs in the small-towns especially in the rural areas, and the nationwide compulsory premarital contraception counseling. To expand the freedom of the press it is essential to increase the legislation and institutions that safeguard the independence of the media by the government and upgrade the training for journalists in the fields of human rights, ethical journalism, quality journalism, and safety. Moreover, immediate intensive actions are needed to achieve the health-related development goals. We analyzed data from 79 countries and measured the effects of 15 factors. To identify the factors that influence HALE, future research should evaluate larger datasets and a wider range of factors.

## Appendix A

**Table 4:** List of the low- and lower-middle-income countries by geographical region^a^ (*N = 79*)

Regions	*n*	Countries	
		Low-income	Lower-middle-income
Africa	43		
Eastern Africa	15	Burundi, Comoros, Eritrea, Ethiopia, Madagascar, Malawi, Mozambique, Rwanda, Somalia, Uganda, United Republic of Tanzania, Zimbabwe	Djibouti, Kenya, Zambia
Middle Africa	6	Central African Republic, Chad, Democratic Republic of the Congo	Cameroon, Congo, Sao Tome and Principe
Northern Africa	4	South Sudan	Egypt, Morocco, Sudan
Southern Africa	2		Lesotho, Swaziland
Western Africa	16	Benin, Burkina Faso, Gambia, Guinea, Guinea-Bissau, Liberia, Mali, Niger, Sierra Leone, Togo	Cabo Verde, Côte d’Ivoire, Ghana, Mauritania, Nigeria, Senegal
Oceania	6		Kiribati, Micronesia, Papua New Guinea, Samoa, Solomon Islands, Vanuatu
Asia	21		
South Asia	7	Afghanistan, Nepal	Bangladesh, Bhutan, India, Pakistan, Sri Lanka
South-East Asia	7	Cambodia	Indonesia, Lao People’s Republic, Myanmar, Philippines, Timor-Leste, Vietnam
Western Asia	4		Armenia, Georgia, Syrian Arab Republic, Yemen
Central Asia	3		Kyrgyzstan, Tajikistan, Uzbekistan
America and the Caribbean	7		
Central America	4		El Salvador, Guatemala, Honduras, Nicaragua
South America	2		Bolivia, Guyana
The Caribbean	1	Haiti	
Europe	2		Republic of Moldova, Ukraine

Notes: ‘*n*, Number of countries’; ‘^a^, Based on the United Nation’s geographical region’ (reference from [33])

## Appendix B

**Table 5:** Names, brief descriptions and sources of the independent variables

Name of the independent variables	Definition of the independent variables	Sources
Demographic factors		
Total fertility rate	The average number of births reported during a woman’s reproductive life.	[18]
Health related factors		
Millennium development goals (MDGs)	The MDGs are eight international development goals that all 194 United Nations member states and at least 23 international organizations have agreed to achieve by the year 2015.	[15]
Human immunodeficiency virus (HIV) prevalence rate	The percentage of people aged 15–49 who are infected with HIV.	[18]
Physician density	The number of physicians per ten thousand populations.	[15]
Socioeconomic status		
Mean years of schooling	The average number of schooling per person aged 25 and older.	[16]
Gross national income (GNI) per capita	The GNI in purchasing power parity divided by mid-year population.	[18]
Health expenditure	The sum of public and private health expenditures as a ratio of total population.	[18]
Urban population (% of total)	According to National Statistical Offices, the urban population refers to that person who is living in urban areas. To calculate it the necessary information is population estimated by World Bank and urban ratios from the United Nations World Urbanization Prospects.	[18]
Social structural factors		
Freedom of the press	The degree of media freedom analyzing the events and developments of each calendar year. It ranges from 0 (the most free) to 100 (the least free).	[21]
Corruption perceptions index	The perceived levels of corruption in public sector worldwide. It ranges from 0 (highly corrupt) to 100 (very clean).	[19]
Prison population rate	The prison population per 100,000 national populations.	[22]
Economic freedom	The degree of freedom of the people in economic activities which captures various institutional aspects influencing economic activities, based on rule of law, limited government, regulatory efficiency, and open markets. It ranges from 0 (minimum freedom) to 100 (maximum freedom).	[20]
Environmental issues		
Improved drinking-water sources using rate	The percentage of the population using an improved drinking-water source. It includes piped household water connection located inside the user’s dwelling, plot or yard and other improved drinking water sources like public taps or standpipes, tube wells or boreholes, protected dug wells, protected springs, and rainwater collection.	[3]
Improved sanitation using rate	The percentage of the population using an improved sanitation system, where sanitation refers the provision of facilities and services for the safe disposal of human urine and feces.	[3]
Carbon dioxide (CO_2_) emission per capita (tones)	The discharge of CO_**2**_ from the burning of fossil fuels and the production of cement.	[18]

## Abbreviations

CO_**2**_: Carbon Dioxide
CPI: Corruption Perception Index
FH: Freedom House
Fig.: Figure
GNI: Gross National Income
HALE: Healthy Life Expectancy
HF: Heritage Foundation
HIV: Human Immunodeficiency Virus
LE: Life Expectancy
MDGs: Millennium Development Goals
PRB: Population Reference Bureau
TFR: Total Fertility Rate
TI: Transparency International
UN: United Nations
UNDP: United Nations Development Program
US: United States
USA: United States of America
WB: World Bank
WHO: World Health Organization
